# Predicting subsequent task performance from goal motivation and goal failure

**DOI:** 10.3389/fpsyg.2015.00926

**Published:** 2015-07-02

**Authors:** Laura C. Healy, Nikos Ntoumanis, Brandon D. Stewart, Joan L. Duda

**Affiliations:** ^1^Department of Physical Education and Sports Studies, Newman UniversityBirmingham, UK; ^2^Health Psychology and Behavioural Medicine Research Group, School of Psychology and Speech Pathology, Curtin University, PerthWA, Australia; ^3^School of Psychology, College of Life and Environmental Sciences, University of BirminghamBirmingham, UK; ^4^School of Sport, Exercise and Rehabilitation Science, College of Life and Environmental Sciences, University of BirminghamBirmingham, UK

**Keywords:** goal pursuit, self-concordance, self-determination theory, executive function, physical performance

## Abstract

Recent research has demonstrated that the cognitive processes associated with goal pursuit can continue to interfere with unrelated tasks when a goal is unfulfilled. Drawing from the self-regulation and goal-striving literatures, the present study explored the impact of goal failure on subsequent cognitive and physical task performance. Furthermore, we examined if the autonomous or controlled motivation underpinning goal striving moderates the responses to goal failure. Athletes (75 male, 59 female, *M*age = 19.90 years, SDage = 3.50) completed a cycling trial with the goal of covering a given distance in 8 min. Prior to the trial, their motivation was primed using a video. During the trial they were provided with manipulated performance feedback, thus creating conditions of goal success or failure. No differences emerged in the responses to goal failure between the primed motivation or performance feedback conditions. We make recommendations for future research into how individuals can deal with failure in goal striving.

## Introduction

Goals form an important function in daily life. A wealth of research has examined how individuals can optimally strive toward their goals in order to experience goal attainment. However, while individuals may hope for success in all of their endeavors, the reality is that they will, at times, not reach the targeted objective and experience failure in their goal pursuits. In this study we explore how the motivation underpinning goal striving might predict an individual’s responses to goal success or failure.

Carver and Scheier (2003) suggested that individuals may respond in several ways when they appraise the obstacles experienced in goal pursuit as too difficult to overcome. One option is to give up effort, yet remain committed to the goal. Carver and Scheier (2003) proposed that this would lead to feelings of distress and helplessness. Conversely, individuals may disengage from an unattainable goal. Specifically, individuals might choose an alternative path to their goal or form a new goal, both of which might lead to a higher order goal. Alternatively, individuals can scale back their original goal. Both options have the potential for positive behavioral and affective outcomes. Individuals may also disengage from their goal without adopting a new goal. Carver and Scheier (2003) suggested that this latter option would result in aimlessness, emptiness, and loneliness.

As demonstrated in Carver and Scheier’s (2003) framework, goal failure can invoke cognitive, affective, and behavioral responses in individuals. Indeed, there is substantial empirical evidence which demonstrates that goal pursuit can have an impact on cognitive processes. James (1890) first suggested that goals can occupy cognitive resources. Since his original suggestion, there has been extensive research on cognitive processes that are beneficial for goal striving. For example, Locke and Latham (2002), and Moskowitz (2002) suggested that goals direct attention. When engaged in goal striving, individuals access task-relevant knowledge from memory in order to adopt the most appropriate approach to goal pursuit (Locke and Latham, 2002). Furthermore, Shah et al. (2002) demonstrated that conscious processes are important for individuals to shield important goals from other goals, which can facilitate progress in goal pursuit.

It has been suggested that even when an individual fails to achieve a goal, that goal can remain active in working memory for extensive periods of time (Jostmann and Koole, 2009). Additionally, goal failure is often associated with rumination (Martin and Tesser, 1989; Ntoumanis et al., 2014b), and as previously mentioned individuals might remain cognitively engaged in goal pursuit even if they have ceased working toward it (Carver and Scheier, 2003). This may present a problem for goal striving, as attentional resources may be consumed by the initial failed goal instead of new pursuits which remain achievable. Lewin (1935) suggested that goals which have not been achieved can remain active in working memory. Additionally, it has been proposed that such goals can interrupt an individual’s thoughts and attention (Zeigarnik, 1927). Recent research by Masicampo and Baumeister (2011) investigated these notions by exploring how the cognitive processes associated with goal striving might have a negative effect by continuing to occupy cognitive resources when goals are unfulfilled. Unfulfilled goals were operationalized as an objective that an individual had been working toward but was yet to be achieved. As such, Masicampo and Baumeister (2011) suggested that the presence of unfulfilled goals would impact executive function. This has been defined as “a higher order cognitive ability that controls basic and underlying cognitive function for purposeful, goal-directed behavior” (Etnier and Chang, 2009, p. 470). Executive function is involved in the selection, scheduling and coordination of complex cognitive function, including inhibition, planning and cognitive flexibility (Hillman et al., 2008).

In a series of studies, Masicampo and Baumeister (2011) induced unfulfilled goal conditions before asking participants to complete a variety of tasks related to two elements of executive function: fluid intelligence (the ability to maintain and manipulate information in working memory) and impulse control (the selective avoidance of certain stimuli and prevention of prepotent responses to such stimuli). In all studies, the presence of an unfulfilled goal (as opposed to no goal or a fulfilled goal) resulted in poorer performance in the executive function tasks. This effect was moderated by an individual’s goal tenacity disposition (i.e., the degree to which individuals generally persist in pursuing goals all the way to completion), whereby those high in goal tenacity were most impacted by unfulfilled goals. Furthermore, when participants had an unfulfilled goal which was later completed, the negative effect on executive function was no longer evident.

The work of Masicampo and Baumeister (2011) demonstrates that failing to achieve a goal may have consequences that extend beyond this failure. There are, however, some aspects of their work which could be extended. First, in their work, the unfulfilled goal was an aim that was yet to be achieved, in their own words a “frustrated goal.” It could be, therefore, that participants felt they could still fulfill the goal at a later time. However, individuals often experience goal failure without the opportunity to continue working on it. For example, an athlete may set a goal to reach the final of their sport at the next Olympics; however, due to a false start they fail to qualify from the initial heats. While they may be able to adjust the timescale of their goal (e.g., aim to reach the finals at their next major international competition), the opportunity to achieve their original goal is unavailable. To the best of our knowledge, the impact of unattainable goals on executive function has not been addressed within the literature. Given that goals which have not been achieved can intrude on an individual’s thoughts and attention (Zeigarnik, 1927; Lewin, 1935), we might expect that failed goals would have a negative effect on executive function.

While Masicampo and Baumeister (2011) showed that an unfulfilled goal only hindered performance on tasks requiring executive function (Study 3), the goal-related as well as the subsequent tasks were mostly cognitive in nature. It is not known whether these results generalize to physical tasks (primary and secondary) which may not be reliant on executive function. Furthermore, research has not explored how failing in a goal which requires physical exertion impacts performance in subsequent tasks requiring executive function. In the current study the primary task required physical exertion; we also had both cognitive and physical follow-up tasks.

One context where goal striving frequently involves physical exertion is sport; an achievement-driven environment where the setting, pursuit and regulation of goals is commonplace (Weinberg, 2013). Given the prevalence and salience of goal pursuit in sport, it seems important to understand how goal failure might impact subsequent physical performance. Anecdotal evidence tells us that some athletes can perceive failure as beneficial for future performance (for example, in a famous advert Michael Jordan describes his performance failures as the reason for his overall and considerable success). Therefore, we manipulated feedback during a physical task to induce goal success or failure, following which we explored the impact of the feedback on performance in several tasks requiring either execution function or physical performance.

A further way in which the extant literature could be advanced is by investigating the role of other individual differences, besides goal tenacity disposition, on the responses to goal failure. One such individual difference from the wider goal striving literature is the underlying motivation with which people strive for their goals. There is growing evidence that demonstrates how diverse types of motivation can differentially impact goal self-regulation (Sheldon and Elliot, 1999; Smith et al., 2007; Ntoumanis et al., 2014a,b). As such, it may be that differences in goal motivation can either accentuate or diminish the impact of failed goals on subsequent tasks requiring executive function.

According to Sheldon and Elliot (1999), and reflecting the motivational regulations outlined in self-determination theory (SDT; Deci and Ryan, 1985, 2000), goal motives can be split into two broad categories. Autonomous motives, reflecting intrinsic and identified regulations, are aligned with an individual’s personal values, and reflect the perceived enjoyment, challenge or importance of the goal. Controlled goal motives reflect introjected and extrinsic regulations and are the products of pressure, which may be from external sources (such as the expectations of important others) or internal factors (for example, feelings of guilt). When striving is underpinned by autonomous motives, individuals have a greater sense of volition, which has been identified as a key determinant of effort in goal striving (Gollwitzer, 1990). As a result, autonomous goal motives have consistently been linked with a range of positive outcomes, including greater persistence in goal pursuit (Ntoumanis et al., 2014a), higher levels of goal attainment (Sheldon and Elliot, 1999; Smith et al., 2007; Koestner et al., 2008), and greater psychological and physical well-being (Smith et al., 2007, 2010, 2011; Healy et al., 2014). In contrast, controlled goal motives have generally been found to be unrelated to goal persistence and attainment (Smith et al., 2007; Ntoumanis et al., 2014a), and to be negatively or unrelated to well-being (Smith et al., 2007; Healy et al., 2014).

Given these previous findings regarding goal motives and goal-related outcomes, we might expect that goal motives would moderate the impact of failed goals on executive function. Indeed, there may be differences in the responses to goal failure between those striving with autonomous motives, and those who are pursuing goals with controlled motivation. Regarding striving with controlled goal motives, Ntoumanis et al. (2014b) demonstrated that when a goal becomes unattainable, individuals striving with controlled goal motives do not report adaptive self-regulatory responses. Specifically, in two studies, these authors showed that controlled goal motives were unrelated to both cognitive disengagement and reengagement. Given this finding, we expect that there would be no impact on executive function following goal failure when individuals are striving with controlled motivation.

In contrast, there are several reasons why we expect autonomous goal motives to moderate the responses to goal failure. Masicampo and Baumeister (2011) showed that unfulfilled goals have a greater impact on subsequent task performance when individuals reported higher levels of goal tenacity. Given previous findings indicate that those with higher autonomous motives demonstrate greater persistence (Ntoumanis et al., 2014a), it could be argued that there will be negative consequences when individuals fail to achieve a goal for which they are striving with autonomous motives. This notion is supported by recent research which found that athletes with autonomous goal motives struggled to cognitively disengage from a goal which had become unattainable (Ntoumanis et al., 2014b). It may be that a similar effect is shown in the responses to goal failure and as such, it was hypothesized that autonomous goal motives will have a negative impact on executive function resources when individuals experience goal failure.

Equally, however, we could provide an argument as to why autonomous goal motives might be beneficial following goal failure. While Ntoumanis et al. (2014b) found that those with autonomous motives struggled to disengage from an unattainable goal, they also showed that these individuals found it easier to cognitively reengage in an alternative goal (which led to the same higher order goal), as long as they realized early in their striving that the goal had become unattainable. Furthermore, autonomous goal motives have consistently been associated with greater positive affect (Smith et al., 2007; Ntoumanis et al., 2014a), which has been shown to play an important role in promoting goal flexibility (Marien et al., 2012). Given these findings, we expected that there would be less of a negative effect on executive function when individuals fail to achieve a goal which is underpinned by autonomous motives.

To summarize, the aim in the present investigation was to examine how goal motivation might moderate (by augmenting or buffering) the impact of goal failure on post-task executive function and subsequent physical task performance. We expected that there would be no differences in the outcome variables between autonomous and controlled goal motives when the goal was achieved, but a moderation effect would be evident under goal failure conditions. Specifically, we expected a null effect for controlled motives and a significant effect (either positive or negative, as we had equally plausible competing hypotheses) for autonomous motives under conditions of goal failure.

## Materials and Methods

### Participants

Following ethical approval from the Science, Technology, Engineering and Mathematics Ethical Review Committee at the University of Birmingham, we recruited 136 athletes (75 male, 59 female, *M*age = 19.90 years, SDage = 3.50) from various sports (except cycling and triathlon to avoid inclusion of participants with experience in cycling events) in return for course credit or financial reward (£5). These athletes were from a variety of team (e.g., netball, hockey, rugby) and individual (e.g., athletics, boxing, and swimming) sports, and trained on average for 5.98 h every week (SD = 4.07). All participants were aged 18 or over, and were informed they could withdraw from the study without being required to provide a reason for their withdrawal. Written informed consent was gained from all participants prior to participation. An a priori power analysis conducted using GPower 3.1 (Faul et al., 2007) based on an effect size of *f* = 0.15 indicated that a sample of 128 participants were needed for α = 0.05 and power = 0.80.

### Design

We used a 2 (outcome condition: success/failure) by 2 (prime condition: autonomous/controlled) between-subjects design. Participants were randomly assigned to one of four experimental conditions: autonomous prime success feedback (AS; *n* = 36), autonomous prime failure feedback (AF; *n* = 33), controlled prime success feedback (CS; *n* = 32) and controlled prime failure feedback (CF; *n* = 35). Our primary goal task involved an 8-min trial during which participants had to cover an individually assigned distance goal on a cycle ergometer. We were primarily interested in the impact of our experimental conditions on secondary task performance. As such, we used three secondary tasks, which participants performed in a randomly assigned order following the cycling trial. Of these three tasks, two measured executive function [Trail Making Test (TMT) and Anti-Saccade Test (AST)] and one assessed physical performance.

### Measures

#### Motivational Primes

In order to examine the impact of different goal motives on the responses to goal failure, participants were exposed to either an autonomous or controlled motivation video prime. These primes consisted of watching a gender-matched actor describing their motivation for an upcoming unrelated to our study task, and were used to induce the goal motivation for the necessary condition. The primes were presented on a computer screen and lasted between 2:14 and 2:45 min (depending on the gender and the condition). We developed these primes specifically for goal motives research. In the autonomous prime, the actor described striving for an unrelated goal because of the personal importance of the goal, and how the goal would be challenging but enjoyable. Conversely, within the controlled prime the actor portrayed that they were striving to avoid guilt-related feelings. The primes have been shown to invoke behavioral responses in accordance with Sheldon and Elliot’s (1999) theoretical model, whereby individuals exposed to the autonomous prime demonstrated greater goal persistence than those who received the controlled prime (Ntoumanis et al., 2014a). As a cover story, and consistent with Ntoumanis et al. (2014a), participants were informed that the primes formed part of a separate, unrelated study exploring the impact of exercise on memory. During the funneled debriefing participants completed items pertaining to the goal motivation of the actor in the prime, to ensure that the primes were perceived in the manner intended. We administered four items (e.g., “*To what extent did the participant in the video suggest that they were going to try and achieve their goal to avoid feeling guilty?*”; *“To what extent did the participant in the video suggest that they expected to enjoy the activity they were about to do?”*) which reflected either controlled or autonomous goal motives. These items were presented as memory questions in order to maintain our cover story, and participants rated them on a 1 (*not at all*) to 7 (*very much so*) scale.

#### Main Task

During the main 8-min cycling trial, we displayed manipulated feedback to participants on a computer screen immediately in front of the cycle ergometer. This was updated every minute to provide the participant with information related to their progress toward their goal, and varied dependent on the experimental condition. Participants in the AS and CS conditions received feedback to suggest that they were making better than expected progress, with the final values showing they had achieved their goal as they had covered a distance greater than their goal (attaining between 108 and 111% of their original goal). Participants in the AF and CF conditions received feedback to suggest that they were making worse than expected progress with the final values showing they had not achieved their goal (reaching between 89 and 92% of their target distance).

#### Secondary Task Performance

##### Trail making test

The TMT measures cognitive abilities such as visual scanning with a motor component, cognitive flexibility and task-set inhibition ability (Etnier and Chang, 2009). TMT consisted of two parts. In part A, participants were required to sequentially link 25 encircled numbers (e.g., 1, 2, 3, 4, etc.) on a sheet of paper as quickly as possible. Part B followed a similar format; however, the sequence included alternating numbers and letters (e.g., 1, A, 2, B, 3, C, etc.). Participants were given a shorter version of both parts in order to familiarize themselves with the task prior to performing the actual test. For each participant, the time to complete the TMT was recorded on a stopwatch by an experimenter. Participants were required to correctly complete the TMT; if there were any mistakes then the time continued while they returned to make corrections before fully completing the task. For the purpose of the analyses, the time to complete Part A was subtracted from the time to complete part B to a single dependent variable (TAB cost), as this can isolate the executive function from other lower cognitive abilities (Etnier and Chang, 2009).

##### Anti-saccade test

The AST assesses working memory (Kane et al., 2001). Participants performed the AST, which was designed according to descriptions in previous research (e.g., Everling and Fischer, 1998; Kane et al., 2001), on a computer. Participants were asked to correctly identify a letter (*H* or *T*), briefly presented on the screen by pressing the respective key on a standard UK keypad. These letters were presented in peripheral vision for a period of 100 ms, preceded by a green circle which appeared for 400 ms as an initial preparatory stimulus. The cue and the stimuli were in 20 point font (∼6 mm height × 5 mm width), and were presented at 10.5° of visual angle from the fixation point (+ at the center of the screen). In the first condition, the pro-saccade condition, both the preparatory stimulus and the letter appeared on the same side of the screen. In the anti-saccade condition, the initial stimulus and letter appeared on opposite sides of the screen; thus participants were required to inhibit the response of attending to the initial stimulus in order to correctly identify the letter. The fixation cross (“+”) was presented in the center of the screen for 2000 ms and each condition contained a total of 48 trials. For the purpose of our analysis we used anti-saccade error (the number of incorrect responses made in the anti-saccade condition) as the dependent variable, given that higher anti-saccade error indicates lower cognitive control (Everling and Fischer, 1998).

##### Physical performance

The final measure of subsequent performance was a test of physical performance. We wanted a task for which (a) participants were likely to be familiar with without being trained or experts in the physical movements, and (b) executive function was not a key requirement for successful performance. With this in mind we chose a buzzer task, where participants were required to move a metal wand along a piece of metal wire which had been manipulated to include curves and bends. If the wand touched the wire, an electrical circuit was completed which set off a buzzer sound. Participants were instructed that they had to move the wand from one end of the wire to the other as quickly as possible, while also trying to make as few mistakes as possible. They were also informed that for every time the buzzer sounded, 5 s would be added to their overall time. As such, both speed and accuracy were important for a successful performance of the task. Participants performed this task three times; the time to complete each trial was recorded by the experimenter using a stopwatch. We created a mean of the three trials to use in our analyses.

#### Control Variables

Given that goal striving can be impacted by perceptions of goal difficulty, importance, and efficacy (Locke and Latham, 2002), we asked participants to rate their perceptions of these variables prior to the 8-min cycling trial. Specifically, they completed three items for goal difficulty (e.g., “How challenging is your goal?”), importance (e.g., “How important is it to you that you achieve your goal?”), and efficacy (e.g., “How confident are you that you will achieve your goal?”) on a 1 (*not at all)* to 7 (*very)* scale. During the trial, we asked participants to rate their goal attainment expectancy (e.g., *“To what degree do you believe you are going to achieve your goal?*”) on a 1 (*not at all)* to 7 (*very much)* scale. This was used to ensure that the feedback created the expected perceptions of success and failure for the respective conditions. Every 2 min during the trial, participants rated their perceptions of effort and goal attainment expectancy. The latter was primarily used to ensure that the feedback was perceived in the manner we intended (i.e., those in the failure conditions would have report lower goal attainment expectancy than those in the success conditions).

### Procedure

The experimental protocol was similar to that used in previous goal motives research (e.g., Ntoumanis et al., 2014a,b). Participants completed one individual experimental session. Prior to their arrival in the laboratory they were asked to avoid strenuous exercise for 24 h, and food, alcohol, caffeine, and tobacco for 3 h. Participants were fitted with a heart rate (HR) monitor on arrival to record resting HR, before completing consent forms, a health screening questionnaire, and demographic questions. As a cover story, participants were informed that they would be completing a battery of tests with specific goals which assessed factors important for sport performance; as such it was considered that a higher order goal was to perform well across all tasks.

Participants first completed a warm up on the cycle ergometer, followed by an incremental submaximal test. This was performed in order to standardize the workload across participants, and control for the impact of exercise intensity on their psychological responses (Ekkekakis, 2003). The submaximal test consisted of four 2-min stages where the workload increased at every stage. HR was recorded at the end of each stage, and we extrapolated these values against the workload on the bike. This enabled us to determine the workload required for participants to be working at 50% of their age-predicted maximum HR (220 beats per minute minus age). The load on the bike was set at this level for the 2- and 8-min cycling tasks.

Participants next completed a 2-min cycling trial, which was used to create a personal goal for the main cycling trial. For this task, they were informed that their goal was to cover as much distance as possible. In a rest period following the 2-min trial, participants were informed that the distance they had just covered would be used to calculate their goal for an 8-min cycling trial. Specifically, the 2-min distance was multiplied by four and then slightly adjusted so that the 8-min goal constituted 95% of this multiplicative value. Previous work (Ntoumanis et al., 2014b, Study 2) suggests that this procedure is successful in ensuring the participants feel the goal is difficult yet attainable. Once they were aware of their goal, participants were asked to complete measures that assessed variables which we controlled for in subsequent analyses (e.g., perceptions of goal difficulty, efficacy and importance). The 8-min goal trial then commenced, with the manipulated feedback presented to all participants as previously described.

Following the 8-min trial the participants were presented with the three subsequent tasks, the order of which was randomized across participants. After they had performed all three tasks, participants completed a funneled debriefing (Bargh and Chartrand, 2000) to probe for suspicion of both the motivation prime and the success or failure feedback presented during the cycling trial. Debriefing was completed via email once data had been collected from all participants.

### Data Analysis

Factorial analyses of variance (ANOVAs) and multivariate analyses of variance (MANOVAs) were conducted on the demographic and control variables. There were two between-subject factors; outcome condition (success/failure) and prime condition (autonomous/controlled). In order to ensure that the primes and feedback were perceived in the manner expected across the experimental conditions, we performed two manipulation checks. First, we conducted a mixed model ANOVA for the participants’ goal attainment expectancy during the trial. Again, the between-subject factors were the prime and outcome conditions, and the within-subject factor was time (2-, 4-, and 6-min). Of particular interest was the change in perceptions of goal attainment over the trial, and how this was predicted by the prime by outcome interactions. For this analysis, the Greenhouse-Geisser epsilon correction was employed if the Maulchly’s test indicated that the assumption of sphericity had been violated. Additionally, to ensure the primes had been perceived as we expected, a factorial (prime condition × outcome condition) MANOVA was conducted on participants’ responses to the items regarding the actor’s goal motivation. Our primary analyses relating to secondary task performance were analyzed using factorial ANOVA. For all analyses, the alpha level was set at *p*< 0.05.

## Results

### Preliminary Analyses

Six participants who indicated suspicion of either the prime or the success/failure feedback were removed from all analyses. The data were screened for multivariate outliers using Mahalanobis distance; this resulted in the removal of four further participants. Hence, the final sample consisted of 126 participants (AS; *n* = 34, AF; *n* = 31, CS; *n* = 32, CF; *n* = 29).

We present the descriptive statistics and scale reliabilities in **Table 1**. We first conducted preliminary tests to ensure that our findings would not be confounded by group differences in demographics or control variables. The findings of these preliminary analyses are displayed in **Table 2**. Separate two (outcome condition: success/failure) by two (prime condition: autonomous/controlled) factorial ANOVAs showed no significant main effects or interactions for any of the demographic or control variables. There were also no significant main effects or interactions on the actual total distance covered by participants (as opposed to the distance displayed by the manipulated feedback). A two (outcome condition: success/failure) by two (prime condition: autonomous/controlled) multivariate analysis of variance (factorial MANOVA) revealed no multivariate or univariate main effects or interactions for the goal-related variables (goal difficulty, goal efficacy, goal importance). Taken together, the non-significant findings of these analyses suggest that our results were not confounded by group differences in demographics or control variables.

**Table 1:** Cronbach’s internal reliabilities and descriptive statistics for study variables by condition.

		AS	AF	CS	CF
	α	*M* (SD)	*M* (SD)	*M* (SD)	*M* (SD)
Goal difficulty	0.93	5.03 (1.00)	5.42 (0.85)	5.23 (1.05)	5.01 (1.07)
Goal efficacy	0.93	4.97 (1.23)	4.65 (1.18)	4.72 (1.03)	4.95 (1.13)
Goal importance	0.88	5.03 (1.04)	5.02 (0.88)	4.93 (1.38)	5.28 (1.04)
Actor autonomous goal motives	0.77	6.38 (0.72)	6.00 (1.31)	2.66 (1.17)	2.86 (1.32)
Actor controlled goal motives	0.71	3.28 (1.58)	3.23 (1.47)	6.50 (0.94)	6.69 (0.47)
TMT Part A	–	26.46 (6.74)	23.72 (6.60)	24.42 (7.73)	24.35 (8.24)
TMT Part B	–	56.08 (37.69)	46.25 (18.22)	48.01 (15.79)	50.18 (18.16)
AST	–	0.06 (0.04)	0.04 (0.04)	0.05 (0.05)	0.06 (0.04)
Buzzer task	–	69.86 (29.76)	80.33 (32.99)	73.65 (28.84)	70.21 (26.03)

*AS, autonomous success; AF, autonomous failure; CS, controlled success; CF, controlled failure; TMT, trail making test; AST, anti-saccade test.*

**Table 2:** Results of analyses of variance (ANOVAs) and multivariate analyses of variance (MANOVAs) conducted as preliminary analyses and manipulation checks.

	*df*	Pillai’s *V*	*F*	*p*	η^2^
Age					
Outcome	1, 122	–	1.30	0.26	0.01
Prime	1, 122	–	0.06	0.81	<0.001
Outcome × Prime	1, 122	–	3.13	0.08	0.001
Hours of training					
Outcome	1, 122	–	0.17	0.68	0.01
Prime	1, 122	–	0.008	0.93	<0.001
Outcome × Prime	1, 122	–	0.01	0.91	<0.001
Hours of cycling					
Outcome	1, 122	–	2.10	0.15	0.01
Prime	1, 122	–	0.24	0.62	0.002
Outcome × Prime	1, 122	–	0.16	0.69	0.001
Distance in 2-min cycling trial					
Outcome	1, 122	–	0.01	0.83	<0.001
Prime	1, 122	–	0.01	0.91	<0.001
Outcome x Prime	1, 122	–	3.01	0.09	0.02
Distance in 8-min cycling trial					
Outcome	1, 122	–	0.42	0.52	0.003
Prime	1, 122	–	0.07	0.80	0.001
Outcome × Prime	1, 122	–	2.08	0.15	0.02
Goal-related variables					
Multivariate effects					
Outcome	3, 120	0.01	0.43	0.73	0.01
Prime	3, 120	0.01	0.40	0.75	0.01
Outcome × Prime	3, 120	0.03	1.12	0.34	0.03
Goal difficulty					
Outcome	1, 122	–	0.77	0.38	0.006
Prime	1, 122	–	0.61	0.44	0.005
Outcome × Prime	1, 122	–	2.01	0.16	0.02
Goal efficacy					
Outcome	1, 122	–	0.02	0.89	<0.001
Prime	1, 122	–	0.05	0.83	<0.001
Outcome × Prime	1, 122	–	1.88	0.17	0.02
Goal importance					
Outcome	1, 122	–	0.17	0.68	0.001
Prime	1, 122	–	0.70	0.40	0.006
Outcome × Prime	1, 122	–	0.87	0.35	0.007
Goal attainment expectancy					
Outcome	1, 122	–	50.55	<0.001	0.29
Prime	1, 122	–	0.32	0.58	0.003
Outcome × Time	1.71, 208.85	–	72.28	<0.001	0.37
Prime × Time	1.71, 208.85	–	0.64	0.53	0.005
Perceptions of actor goal motives					
Multivariate effects					
Outcome	2, 121	0.002	0.13	0.88	0.002
Prime	2, 121	0.79	225.74	<0.001	0.79
Outcome × Prime	2, 121	0.02	1.33	0.27	0.02
Autonomous goal motives					
Outcome	1, 122	–	0.19	0.67	0.002
Prime	1, 122	–	283.41	<0.001	0.70
Outcome × Prime	1, 122	–	2.08	0.15	0.02
Controlled goal motives					
Outcome	1, 122	–	0.10	0.76	0.001
Prime	1, 122	–	236.10	<0.001	0.66
Outcome × Prime	1, 122	–	0.31	0.58	0.003

In addition to our preliminary analyses, we also conducted manipulation checks. These findings are also displayed in **Table 2**. A mixed model ANOVA on participants’ perceptions of goal attainment expectancy indicated a significant time by outcome condition interaction. Pairwise comparisons revealed that those in the success conditions reported higher goal attainment expectancy than those in the failure conditions at all time points. There was also an outcome condition main effect whereby those in the success conditions reported higher overall goal attainment expectancy than those in the failure conditions. There were no other significant main effects or interactions.

A MANOVA examining the participants’ responses to the items regarding the actor’s goal motivation confirmed that the prime had been perceived in the manner we anticipated across the prime conditions. Specifically, there was a significant multivariate main effect for prime condition but no main effect for outcome condition and no interaction Furthermore, there were significant differences in the ratings of the actor’s autonomous and controlled goal motivation between the different primes, with those receiving the autonomous prime rating the actor as higher in autonomous and lower in controlled goal motives than those viewing the controlled prime, and vice versa. Importantly, there were no significant univariate effects for the outcome condition and no interaction. As such, we were satisfied that our primes and the manipulations of feedback had been perceived by participants in line with the four experimental conditions we wished to create.

### Secondary Task Performance

**FIGURE 1:**
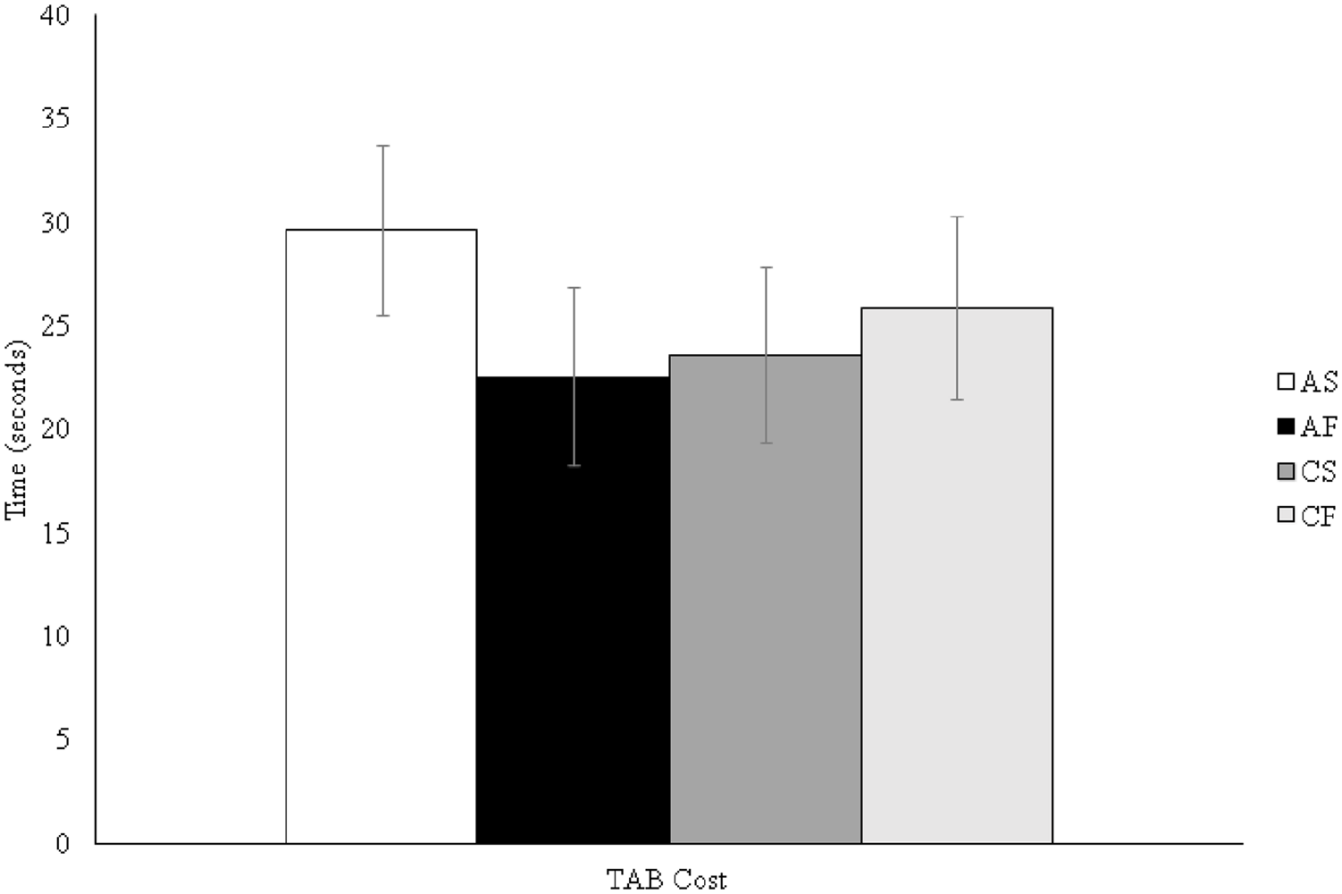
**Trial making test (TMT) TAB cost by experimental condition.** AS, autonomous success; AF, autonomous failure; CS, controlled success; CF, controlled failure.

**FIGURE 2:**
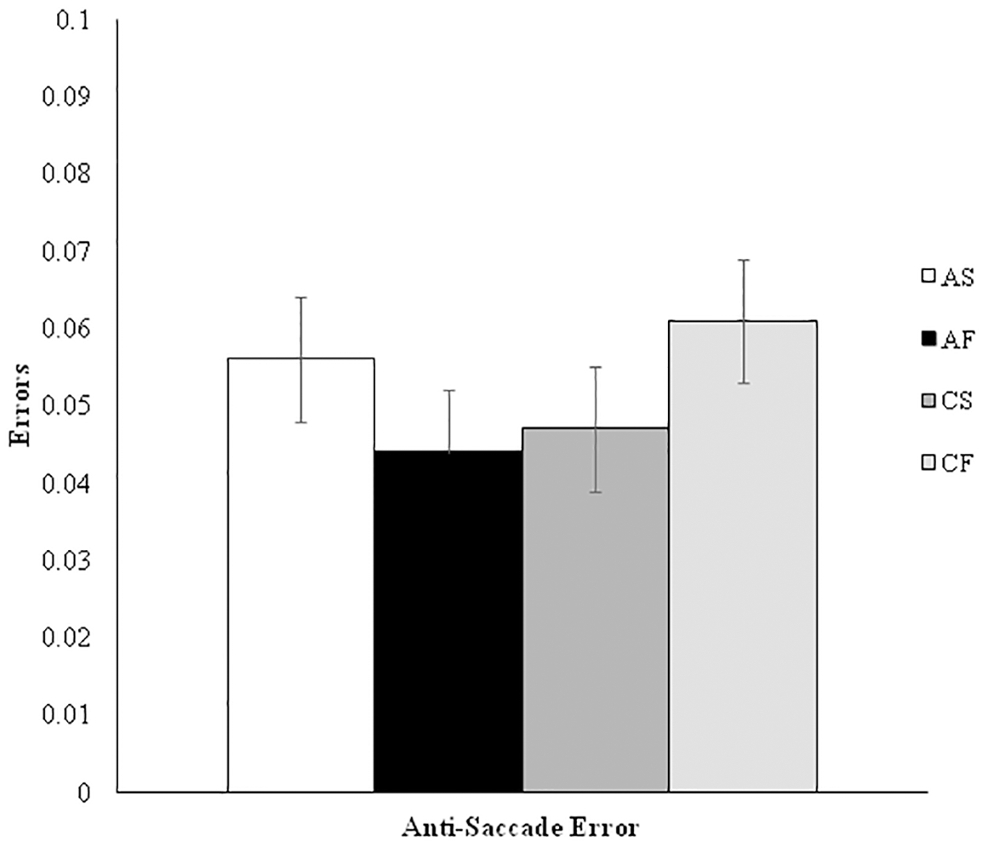
**Anti-saccade error by experimental condition.** AS, autonomous success; AF, autonomous failure; CS, controlled success; CF, controlled failure.

**FIGURE 3:**
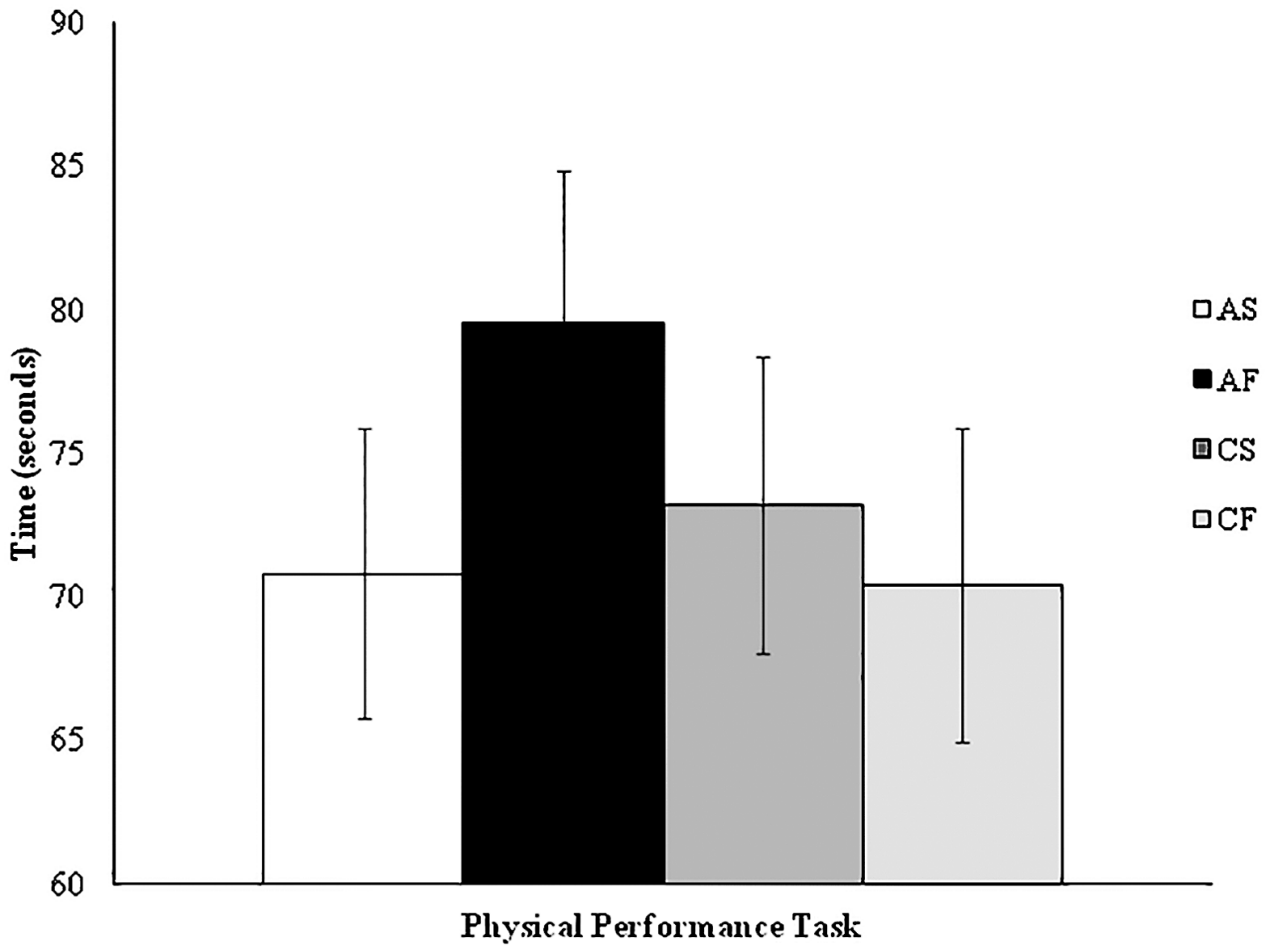
**Physical performance task time by experimental condition.** AS, autonomous success; AF, autonomous failure; CS, controlled success; CF, controlled failure.

For the TMT analysis, we conducted a two (outcome condition: success/failure) by two (prime condition: autonomous/controlled) ANOVA on the TAB cost score. This factorial ANOVA revealed no significant main effects for outcome condition [*F*(1,122) = 0.33, *p* = 0.57, $\eta_{p}^{2}$ = 0.003] or prime condition [*F*(1,122) = 0.10, *p* = 0.75, $\eta_{p}^{2}$ = 0.001], and no interaction [*F*(1,122) = 1.20, *p* = 0.28, $\eta_{p}^{2}$ = 0.01]. These findings are displayed in **Figure 1**. A two (outcome condition: success/failure) by two (prime condition: autonomous/controlled) factorial ANOVA on the anti-saccade error showed there were also no significant main effects for outcome [*F*(1,122) = 0.02, *p* = 0.89, $\eta_{p}^{2}$ < 0.001] or prime condition [*F*(1,122) = 0.29, *p* = 0.59, $\eta_{p}^{2}$ = 0.002], and no interaction [*F*(1,122) = 2.81, *p* = 0.10, $\eta_{p}^{2}$ < 0.02]. A final two (outcome condition: success/failure) by two (prime condition: autonomous/controlled) factorial ANOVA on the physical performance task showed no outcome [*F*(1,122) = 0.45, *p* = 0.51, $\eta_{p}^{2}$ = 0.004] or prime condition [*F*(1,122) = 0.36, *p* = 0.55, $\eta_{p}^{2}$ = 0.003] main effects, and no interaction [*F*(1,122) = 1.74, *p* = 0.19, $\eta_{p}^{2}$ = 0.01]. The AST and physical performance task findings are displayed in **Figures 2** and **3** respectively.

## Discussion

This study sought to investigate the moderating effects of goal motivation on the responses to goal failure. Drawing from the self-regulation (e.g., Masicampo and Baumeister, 2011) and goal striving (e.g., Sheldon and Elliot, 1999) literatures, we hypothesized that under conditions of goal failure there would be an effect of autonomous goal motives on performance in subsequent tasks requiring either executive function or physical movements. Results revealed no support for our hypotheses.

### Goal Failure and Executive Function

Zeigarnik (1927) demonstrated that unfulfilled goals can remain accessible in memory, and therefore can interfere with subsequent tasks. Masicampo and Baumeister’s (2011) work supported this notion. In view of these findings, we expected that, regardless of goal motives, those in the goal failure condition would have performed worse in the executive function tasks than those in the goal success condition. We found no support for this. The differences in how we manipulated goal failure might explain why our findings failed to replicate those of Masicampo and Baumeister (2011). These authors used predominately cognitive tasks to induce an unfulfilled goal; that is a goal which has yet to be achieved. In contrast, within our work we used a physical task where participants experienced failure (or success) in their goal pursuit. It is therefore plausible that when goals are failed, as opposed to not yet being achieved, individuals are left without an opportunity to continue in goal pursuit, and the relevant cognitive processes cease to operate with no impact on post-task executive function. In relation to theoretical perspectives, Zeigarnik (1927) showed that when goals are achieved they cease to occupy cognitive resources. Our results suggest that the same applies when goals are failed, in that there is no impact on executive functions. It may be worthwhile for future research to further explore the consequences of goal failure, goal achievement and unfulfilled goals in relation to secondary tasks requiring executive function.

A further explanation for our null finding is the fact that we used a physical task as our initial goal trial. It may be that the acute exercise performed by participants had an impact on their ability to perform the subsequent tasks. A recent meta-analysis which examined the relationship between exercise and cognitive function showed a small but significant improvement in cognitive function following acute exercise (Lambourne and Tomporowski, 2010). While this meta-analysis did not exclusively look at executive function, it may be that the positive impact of exercise in the present study masks the effect of goal failure on the subsequent executive function tasks. Furthermore, bouts of moderate intensity exercise lasting less than 10 min have been shown to have a positive effect on executive function in healthy adults (Chang et al., 2012). Future studies which wish to explore the impact of unfulfilled or failed goals within a sporting environment may wish to consider using tasks which are not physically exerting or use different exercise durations. For example, goal failure could be manipulated using a discrete skill such as a golf tee shot, and the subsequent impact on related motor skills (e.g., golf putting) and executive function could be tested without the confounding effect of acute exercise.

### Goal Motivation, Goal Failure and Subsequent Task Performance

We expected that the effect of goal failure on subsequent task performance would be moderated by the motives underpinning goal striving. Based on previous literature, we expected that those primed with autonomous (as opposed to controlled) goal motives to either have poorer performance (due to higher levels of goal tenacity; Masicampo and Baumeister, 2011) or greater performance on the subsequent tasks (due to their ability to reengage in alternative goals; Ntoumanis et al., 2014b). Our findings offered no support for either hypothesis, as there were no significant differences in subsequent task performance between any of the experimental groups. To the best of our knowledge, the present study was the first to employ goal motivation priming in relation to post-task performance. Previous studies have found a beneficial effect of primed autonomous goal motives in relation to in-task persistence (Ntoumanis et al., 2014a). Therefore, it is possible that the impact of goal motivation priming following task completion is not as strong as the effect during goal striving. The primes were administered immediately prior to the cycling trial, so it may be that their effect had dissipated by the time of the subsequent trials. In future research, it may be worthwhile re-priming participants prior to the secondary tasks.

### Limitations and Future Research Directions

While the design of the study has several strengths, such as the experimental manipulation of goal motives and goal attainment, and inclusion of both self-report and objective measures, there are also limitations which should be acknowledged. It is possible that the number of tasks involved in the study overloaded the participants. As such, it may be more appropriate for future studies to focus on only one measure of secondary task performance. Further to this, we only manipulated goal motivation for the initial trial. Given that the length of time a priming effect lasts is dependent on the strength of the priming manipulation (Higgins et al., 1985; Bargh and Chartrand, 2000), it may be that the effect of the manipulated motivation diminished following the cycling trial leading to no effects on the subsequent executive function and physical tasks. Our manipulation checks did show that participants could accurately report the actor’s motivation in their respective condition, however, it may be that the motivational strength of the primes had diminished when participants were performing the secondary tasks. It may be worthwhile for future research to consider using an additional related prime for the subsequent tasks in order to retain the motivational impact of such methods.

A further limitation of our work is the lack of clear reengagement opportunities. In a study examining motivation and goal disengagement/reengagement by Ntoumanis et al. (2014b), the secondary task used was a reengagement opportunity which led to the same higher order goal as the initial goal trial. This reflected Carver and Scheier’s (2003) suggestion that reengagement in an alternative goal which leads to the same higher order goal as the initial goal can have positive psychological and behavioral outcomes. Our secondary tasks were not as clearly related to each other as those used by Ntoumanis et al. (2014b).

Given that this was, to the best of our knowledge, the first study to investigate the cognitive responses to goal failure, it is important that future research continues to explore the impact of failed (as opposed to unfulfilled) goals on executive function. There are several areas which may be explored in future research. The most obvious is to address the aforementioned limitations in the current design. It could also involve using an initial goal task which is less physically exerting. It may also be worthwhile to examine how personal goal motives (independently or in conjunction with primed goal motives) impact on responses to goal failure. Future research could also explore how the failure to achieve goals in one important life domain (such as sport) can impact goal pursuit in another domain (such as education or work). The reality of life is that we are continually working toward multiple goals within and across domains (Louro et al., 2007). As such, it is important that research identifies factors which allow optimal goal striving within and across different contexts, particularly when failure is realized in an important life domain. Finally, if exercise improves executive functioning, and if goal failure harms executive functioning, our null findings might suggest that exercise could be a protective factor for executive functioning against goal failure. However, given that this was not the primary aim of the present study it would be worthwhile designing studies specifically to test this suggestion in future research.

## Conclusion

To conclude, the present investigation found no support for hypothesized moderating role of goal motivation in the responses to goal failure. Despite this, we feel that our study is potentially important as the psychological literature is dominated by studies with significant findings (Bakker et al., 2012). Recent papers (Maner, 2014) have highlighted the importance of publishing null findings, particularly where studies fail to replicate existing findings, in order to allow for more comprehensive and balanced future meta-analyses. Our study highlights the need for additional experimental investigations into how the motivation underpinning goal striving may (or may not) relate to how individuals react and adapt when they experience failure in pursuit of important goals.

## Conflict of Interest Statement

The authors declare that the research was conducted in the absence of any commercial or financial relationships that could be construed as a potential conflict of interest.
